# Supplementary material for: Psychological, social, and health-related factors predict risk for financial exploitation
Source: Commun Psychol. 2025 Jun 5;3:88. doi: 10.1038/s44271-025-00266-x (PMC12141040; doi:10.1038/s44271-025-00266-x)
Supplement: Supplementary file 2 — Supplementary Information [file 44271_2025_266_MOESM2_ESM.pdf]

Supplemental Material

Table S1. Study 1, single factor effects of risk factors on OAFEM controlling for age, gender, race, and education level where applicable

Risk Factor			Size	Range	Mean (Std)	Range	Mean (Std)	beta coefficient	f² (effect size)	95% CI	p value	
				OAFEM		Risk Factor						
Sociodemographic factors		Socioeconomic Status	655	0 - 114	5.4 (10.49)	5 - 30	18.97 (5.29)	-0.03	0.000	[-0.002,0.003]	0.058	
Health-related Factors		Physical Health	680	0 - 114	5.41 (10.67)	6 - 20	15.22 (2.84)	-0.13	0.022	[0.007,0.048]	< 0.001	*
		Mental Health	680	0 - 114	5.41 (10.67)	5 - 20	14.52 (3.27)	-0.14	0.019	[0.006,0.039]	< 0.001	*
		Everyday Cognitive Decline	679	0 - 114	5.42 (10.68)	1 - 3.92	1.28 (0.35)	0.16	0.030	[0.011,0.071]	< 0.001	*
Psychosocial Factors	Economic Decision Making Factors	Trust in Strangers	625	0 - 84	5.39 (10.08)	0 - 1	0.43 (0.32)	0.02	0.001	[0,0.006]	0.374	
		Trust in Friends	625	0 - 84	5.39 (10.08)	0 - 1	0.58 (0.31)	0.01	0.000	[-0.001,0.002]	0.795	
		Trust in Friends vs Strangers	625	0 - 84	5.39 (10.08)	-0.51 - 1	0.15 (0.28)	-0.01	0.000	[0,0.004]	0.451	
		Unfair Offer Rejection Rate	625	0 - 84	5.39 (10.08)	0 - 1	0.41 (0.41)	0.01	0.001	[0,0.007]	0.477	
		Fair Offer Rejection Rate	625	0 - 84	5.39 (10.08)	0 - 1	0.02 (0.12)	-0.03	0.001	[-0.001,0.003]	0.141	
		Rejection Rate, Unfair Offers vs Fair Offers	625	0 - 84	5.39 (10.08)	-0.5 - 1	0.39 (0.41)	0.02	0.001	[0,0.009]	0.242	
	Social and Affective Factors	Perceived Social Support	680	0 - 114	5.41 (10.67)	1 - 7	5.39 (1.21)	-0.10	0.010	[0.003,0.025]	< 0.001	*
		Need to Belong	680	0 - 114	5.41 (10.67)	11 - 49	29.51 (6.53)	0.03	0.001	[-0.001,0.004]	0.093	
		Persuadability	680	0 - 114	5.41 (10.67)	6 - 42	11.33 (5.81)	0.17	0.036	[0.014,0.073]	< 0.001	*
		Insensitivity to trustworthiness	680	0 - 114	5.41 (10.67)	6 - 34	14.55 (5.65)	0.10	0.011	[0.003,0.026]	< 0.001	*
		Extrinsic Affect-Improving	680	0 - 114	5.41 (10.67)	1 - 5	3.49 (0.98)	0.07	0.006	[0.002,0.016]	< 0.001	*
		Extrinsic Affect-Worsening	680	0 - 114	5.41 (10.67)	1 - 5	1.29 (0.54)	0.06	0.004	[0,0.018]	< 0.001	*
		Intrinsic Affect-Improving	680	0 - 114	5.41 (10.67)	1 - 5	3.11 (0.98)	0.06	0.005	[0.001,0.014]	< 0.001	*
		Intrinsic Affect-Worsening	680	0 - 114	5.41 (10.67)	1 - 4.75	1.31 (0.59)	0.13	0.018	[0.005,0.046]	< 0.001	*

* significant after multiple comparison correction, alpha corrected = 0.0025

Table S2, Study 1 SES interactions controlling for age, gender, race, and education level where applicable

* significant after multiple comparison correction, alpha corrected = 0.0025

Risk Factor			Size	Range	Mean (Std)	Range	Mean (Std)	Range	Mean (Std)	interaction beta	f² (effect size)	95% CI	p value	
				OAFEM		SES		Risk Factor						
Health-related Factors		Physical Health * SES	655	0 - 114	5.4 (10.49)	5 - 30	18.97 (5.29)	6 - 20	15.21 (2.87)	0.00	0.000	[-0.003,0]	0.818	
		Mental Health * SES	655	0 - 114	5.4 (10.49)	5 - 30	18.97 (5.29)	5 - 20	14.52 (3.28)	0.02	0.002	[-0.001,0.011]	0.021	
		Everyday Cognitive Decline * SES	654	0 - 114	5.41 (10.49)	5 - 30	18.98 (5.29)	1 - 3.92	1.28 (0.36)	0.15	0.004	[0,0.043]	0.103	
Psychosocial Factors	Economic Decision Making Factors	Trust in Strangers * SES	603	0 - 84	5.35 (9.82)	5 - 30	19.14 (5.26)	0 - 1	0.43 (0.32)	0.09	0.000	[-0.001,0.003]	0.391	
		Trust in Friends * SES	603	0 - 84	5.35 (9.82)	5 - 30	19.14 (5.26)	0 - 1	0.58 (0.31)	0.16	0.001	[0,0.006]	0.164	
		Trust in Friends vs Strangers * SES	603	0 - 84	5.35 (9.82)	5 - 30	19.14 (5.26)	-0.51 - 1	0.14 (0.28)	0.09	0.000	[-0.001,0.003]	0.514	
		Unfair Offer Rejection Rate * SES	603	0 - 84	5.35 (9.82)	5 - 30	19.14 (5.26)	0 - 1	0.42 (0.41)	0.00	0.000	[-0.002,0.001]	0.990	
		Fair Offer Rejection Rate * SES	603	0 - 84	5.35 (9.82)	5 - 30	19.14 (5.26)	0 - 1	0.02 (0.13)	0.65	0.001	[0,0.016]	0.055	
		Rejection Rate, Unfair Offers vs Fair Offers * SES	603	0 - 84	5.35 (9.82)	5 - 30	19.14 (5.26)	-0.5 - 1	0.39 (0.41)	-0.06	0.000	[-0.002,0.001]	0.499	
	Social and Affective Factors	Perceived Social Support * SES	655	0 - 114	5.4 (10.49)	5 - 30	18.97 (5.29)	1 - 7	5.39 (1.21)	0.05	0.002	[-0.001,0.012]	0.112	
		Need to Belong * SES	655	0 - 114	5.4 (10.49)	5 - 30	18.97 (5.29)	11 - 49	29.51 (6.58)	-0.01	0.000	[-0.001,0.003]	0.319	
		Persuadability * SES	655	0 - 114	5.4 (10.49)	5 - 30	18.97 (5.29)	6 - 42	11.29 (5.79)	-0.01	0.001	[-0.001,0.013]	0.227	
		Insensitivity to trustworthiness * SES	655	0 - 114	5.4 (10.49)	5 - 30	18.97 (5.29)	6 - 32	14.47 (5.6)	-0.01	0.001	[-0.001,0.005]	0.019	
		Extrinsic Affect-Improving * SES	655	0 - 114	5.4 (10.49)	5 - 30	18.97 (5.29)	1 - 5	3.49 (0.99)	0.02	0.000	[-0.002,0]	0.552	
		Extrinsic Affect-Worsening * SES	655	0 - 114	5.4 (10.49)	5 - 30	18.97 (5.29)	1 - 5	1.29 (0.54)	0.14	0.002	[0,0.027]	0.053	
		Intrinsic Affect-Improving * SES	655	0 - 114	5.4 (10.49)	5 - 30	18.97 (5.29)	1 - 5	3.11 (0.99)	0.04	0.000	[-0.001,0.004]	0.305	
		Intrinsic Affect-Worsening * SES	655	0 - 114	5.4 (10.49)	5 - 30	18.97 (5.29)	1 - 4.75	1.32 (0.59)	-0.26	0.008	[0,0.095]	< 0.001	*

Table S3, Study 2 single factor effects controlling for age, gender, race, and education level where applicable

* significant after multiple comparison correction, alpha corrected = 0.0034

Risk Factor			Size	OAFEM Range	OAFEM Mean (Std)	Risk Factor Range	Risk Factor Mean (Std)	beta coefficient	f^2 (effect size)	95% CI	p value	
Sociodemographic factor		Socioeconomic Status	304	0 - 62	4.26 (8.61)	1.95 - 49	14 (9.5)	-0.02	0.000	[0,0.005]	0.449	
Health-related Factors		Physical Health	305	0 - 62	4.25 (8.6)	6 - 20	15.38 (3.16)	-0.13	0.017	[0.003,0.056]	< 0.001	*
		Everyday Cognitive Decline	305	0 - 62	4.25 (8.6)	1 - 3.67	1.3 (0.4)	0.18	0.032	[0.002,0.088]	< 0.001	*
Psycho social Factors	Economic Decision Making Factors	Trust in Strangers	305	0 - 62	4.25 (8.6)	0 - 1	0.36 (0.33)	-0.01	0.000	[0,0.002]	0.520	
		Unfair Offer Rejection	305	0 - 62	4.25 (8.6)	0 - 1	0.14 (0.35)	0.01	0.000	[-0.002,0]	0.928	
	Social and Affective Factors	Perceived Social Support	305	0 - 62	4.25 (8.6)	1 - 7	5.32 (1.22)	-0.04	0.001	[0,0.007]	0.040	
		Persuadability	305	0 - 62	4.25 (8.6)	6 - 41	11.41 (6.16)	0.11	0.013	[0.002,0.06]	< 0.001	*
		Insensitivity to trustworthiness	305	0 - 62	4.25 (8.6)	6 - 32	14.71 (5.61)	0.07	0.004	[0,0.017]	< 0.001	*
		Extrinsic Affect-Improving	305	0 - 62	4.25 (8.6)	1 - 5	3.36 (1.09)	0.05	0.002	[0,0.01]	0.022	
		Extrinsic Affect-Worsening	305	0 - 62	4.25 (8.6)	1 - 5	1.29 (0.58)	0.12	0.014	[0.002,0.127]	< 0.001	*
		Intrinsic Affect-Improving	305	0 - 62	4.25 (8.6)	1 - 5	3.07 (1.11)	0.03	0.001	[0,0.006]	0.163	
		Intrinsic Affect-Worsening	305	0 - 62	4.25 (8.6)	1 - 4	1.3 (0.55)	0.09	0.008	[0,0.041]	< 0.001	*

Table S4, Study 2 SES interactions controlling for age, gender, race, and education level where applicable

* significant after multiple comparison correction, alpha corrected = 0.0034

Risk Factor			Size	Range	Mean (Std)	Range	Mean (Std)	Range	Mean (Std)	interaction beta	f^2 (effect size)	95% CI	p value	
				OAFEM		SES		Risk Factor						
Health-related Factors		Physical Health * SES	304	0 - 62	4.26 (8.61)	1.95 - 49	14 (9.5)	6 - 20	15.38 (3.17)	0.047	0.005	[-0.001,0.057]	0.025	
		Everyday Cognitive Decline * SES	304	0 - 62	4.26 (8.61)	1.95 - 49	14 (9.5)	1 - 3.67	1.3 (0.4)	-0.626	0.007	[-0.005,0.099]	< 0.001	*
Psychosocial Factors	Economic Decision Making Factors	Trust in Strangers * SES	304	0 - 62	4.26 (8.61)	1.95 - 49	14 (9.5)	0 - 1	0.36 (0.33)	-0.123	0.000	[0,0.006]	0.471	
		Unfair Offer Rejection * SES	304	0 - 62	4.26 (8.61)	1.95 - 49	14 (9.5)	0 - 1	0.14 (0.35)	-0.002	0.000	[-0.002,0]	0.989	
	Social and Affective Factors	Perceived Social Support * SES	304	0 - 62	4.26 (8.61)	1.95 - 49	14 (9.5)	1 - 7	5.32 (1.22)	-0.030	0.000	[0,0.004]	0.504	
		Persuadability * SES	304	0 - 62	4.26 (8.61)	1.95 - 49	14 (9.5)	6 - 41	11.43 (6.16)	-0.038	0.025	[0,0.273]	< 0.001	*
		Insensitivity to trustworthiness * SES	304	0 - 62	4.26 (8.61)	1.95 - 49	14 (9.5)	6 - 32	14.73 (5.61)	-0.004	0.000	[-0.001,0.008]	0.641	
		Extrinsic Affect-Improving * SES	304	0 - 62	4.26 (8.61)	1.95 - 49	14 (9.5)	1 - 5	3.36 (1.09)	-0.073	0.002	[0,0.014]	0.140	
		Extrinsic Affect-Worsening * SES	304	0 - 62	4.26 (8.61)	1.95 - 49	14 (9.5)	1 - 5	1.29 (0.58)	-0.503	0.051	[0,0.367]	< 0.001	*
		Intrinsic Affect-Improving * SES	304	0 - 62	4.26 (8.61)	1.95 - 49	14 (9.5)	1 - 5	3.06 (1.11)	-0.024	0.000	[-0.001,0.005]	0.601	
		Intrinsic Affect-Worsening * SES	304	0 - 62	4.26 (8.61)	1.95 - 49	14 (9.5)	1 - 4	1.3 (0.55)	-0.565	0.024	[0,0.176]	< 0.001	*

Table S5. Study 3 OAFEM single factors controlling for age, gender, race, and education level where applicable

* significant after multiple comparison correction, alpha corrected = 0.0029

Psychosocial Factor		Size	OAFEM Range	OAFEM Mean (Std)	Risk Factor Range	Risk Factor Mean (Std)	beta	f² (effect size)	95% CI	p value	
Economic Decision Making Factors	Trust in Strangers	844	0 - 115	14.25 (20.5)	0 - 1	0.45 (0.32)	0.03	0.001	[-0.001,0.008]	0.246	
	Trust in Friends	844	0 - 115	14.25 (20.5)	0 - 1	0.66 (0.31)	0.02	0.000	[-0.002,0.004]	0.484	
	Trust in Friends vs Strangers	844	0 - 115	14.25 (20.5)	-0.7 - 1	0.22 (0.32)	-0.01	0.000	[-0.002,0.002]	0.610	
	Unfair Offer Rejection	844	0 - 115	14.25 (20.5)	0 - 1	0.17 (0.38)	0.00	0.000	[-0.002,0.001]	0.987	
Social and Affective Factors	Perceived Social Support	844	0 - 115	14.25 (20.5)	1 - 7	5.04 (1.4)	-0.09	0.011	[0.002,0.026]	< 0.001	*
	Cognitive Reflection	844	0 - 115	14.25 (20.5)	0 - 7	1.72 (1.57)	-0.04	0.005	[0,0.015]	0.070	
	Need to Belong	844	0 - 115	14.25 (20.5)	10 - 49	28.52 (6.73)	0.08	0.010	[0.002,0.022]	0.001	*
	Persuadability	844	0 - 115	14.25 (20.5)	6 - 42	14.16 (7.61)	0.21	0.057	[0.025,0.098]	< 0.001	*
	Insensitivity to Trustworthiness Cues	844	0 - 115	14.25 (20.5)	6 - 36	16.95 (5.98)	0.14	0.035	[0.015,0.061]	< 0.001	*
Socialdemographics & Health-related Moderator	Racial or Ethnic Minority	844	0 - 115	14.25 (20.5)	0 - 1	0.51 (0.5)	-0.04	0.000	[-0.001,0.004]	0.400	
	Gender	844	0 - 115	14.25 (20.5)	0 - 1	0.49 (0.5)	0.05	0.000	[-0.001,0.005]	0.298	
	Socioeconomic Status	844	0 - 115	14.25 (20.5)	3 - 20	11.88 (4.15)	0.07	0.006	[0,0.018]	0.004	
	Everyday Cognitive Decline	835	0 - 115	14.32 (20.58)	1 - 3.5	1.34 (0.42)	0.25	0.087	[0.031,0.146]	< 0.001	*
	Physical Health	844	0 - 115	14.25 (20.5)	5 - 20	15.35 (2.97)	-0.10	0.015	[0.005,0.032]	< 0.001	*
	Mental Health	844	0 - 115	14.25 (20.5)	4 - 20	14.08 (3.64)	-0.07	0.004	[-0.001,0.014]	0.004	

Table S6. Study 3 OAFEM interactions controlling for age, gender, race, and education level where applicable

* significant after multiple comparison correction, alpha corrected = 0.0029

Psychosocial Risk Factor	Socialdemographics & Health-related Moderator		Size	Range	Mean (Std)	Range	Mean (Std)	Range	Mean (Std)	interaction beta	f² (effect size)	95% CI	p value
				OAFEM		Psychosocial Risk Factor		Socialdemographic & Health-related Moderator					
Perceived Social Support	Socio-demographic	Age	844	0 - 115	14.25 (20.5)	1 - 7	5.04 (1.4)	20 - 94	48.06 (19.57)	0.04	0.004	[0,0.022]	0.026
		Gender	844	0 - 115	14.25 (20.5)	1 - 7	5.04 (1.4)	0 - 1	0.49 (0.5)	-0.04	0.000	[-0.004,0.001]	0.948
		Socioeconomic Status	844	0 - 115	14.25 (20.5)	1 - 7	5.04 (1.4)	3 - 20	11.88 (4.15)	0.07	0.001	[-0.001,0.008]	0.371
		Racial or Ethnic Minority	844	0 - 115	14.25 (20.5)	1 - 7	5.04 (1.4)	0 - 1	0.51 (0.5)	0.56	0.000	[-0.001,0.005]	0.391
	Health-related	Everyday Cognitive Decline	835	0 - 115	14.32 (20.58)	1 - 7	5.05 (1.41)	1 - 3.5	1.34 (0.42)	0.20	0.000	[-0.011,0.006]	0.805
		Mental Health	844	0 - 115	14.25 (20.5)	1 - 7	5.04 (1.4)	4 - 20	14.08 (3.64)	0.05	0.001	[-0.001,0.008]	0.562
		Physical Health	844	0 - 115	14.25 (20.5)	1 - 7	5.04 (1.4)	5 - 20	15.35 (2.97)	0.04	0.000	[-0.003,0]	0.667
Need to Belong	Socio-demographic	Age	844	0 - 115	14.25 (20.5)	10 - 49	28.52 (6.73)	20 - 94	48.06 (19.57)	-0.01	0.005	[0,0.019]	0.047
		Gender	844	0 - 115	14.25 (20.5)	10 - 49	28.52 (6.73)	0 - 1	0.49 (0.5)	0.06	0.000	[-0.002,0.002]	0.675
		Socioeconomic Status	844	0 - 115	14.25 (20.5)	10 - 49	28.52 (6.73)	3 - 20	11.88 (4.15)	0.01	0.001	[-0.001,0.008]	0.678
		Racial or Ethnic Minority	844	0 - 115	14.25 (20.5)	10 - 49	28.52 (6.73)	0 - 1	0.51 (0.5)	0.13	0.001	[-0.001,0.007]	0.360
	Health-related	Everyday Cognitive Decline	835	0 - 115	14.32 (20.58)	10 - 49	28.46 (6.71)	1 - 3.5	1.34 (0.42)	0.05	0.000	[-0.004,0.009]	0.783

		Mental Health	844	0 - 115	14.25 (20.5)	10 - 49	28.52 (6.73)	4 - 20	14.08 (3.64)	0.00	0.000	[-0.004,0.002]	0.852	
		Physical Health	844	0 - 115	14.25 (20.5)	10 - 49	28.52 (6.73)	5 - 20	15.35 (2.97)	-0.01	0.000	[-0.002,0.004]	0.781	
Persuadability	Socio-demographic	Age	844	0 - 115	14.25 (20.5)	6 - 42	14.16 (7.61)	20 - 94	48.06 (19.57)	-0.01	0.018	[0.003,0.057]	0.001	*
		Gender	844	0 - 115	14.25 (20.5)	6 - 42	14.16 (7.61)	0 - 1	0.49 (0.5)	0.21	0.002	[-0.002,0.024]	0.095	
		Socioeconomic Status	844	0 - 115	14.25 (20.5)	6 - 42	14.16 (7.61)	3 - 20	11.88 (4.15)	0.04	0.015	[0,0.063]	0.002	*
		Racial or Ethnic Minority	844	0 - 115	14.25 (20.5)	6 - 42	14.16 (7.61)	0 - 1	0.51 (0.5)	0.07	0.000	[-0.003,0.006]	0.586	
	Health-related	Everyday Cognitive Decline	835	0 - 115	14.32 (20.58)	6 - 42	14.07 (7.59)	1 - 3.5	1.34 (0.42)	-0.22	-0.004	[-0.023,0]	0.088	
		Mental Health	844	0 - 115	14.25 (20.5)	6 - 42	14.16 (7.61)	4 - 20	14.08 (3.64)	0.01	0.000	[-0.005,0.024]	0.722	
		Physical Health	844	0 - 115	14.25 (20.5)	6 - 42	14.16 (7.61)	5 - 20	15.35 (2.97)	-0.01	0.001	[-0.001,0.011]	0.467	
Insensitivity to Trustworthiness Cues	Socio-demographic	Age	844	0 - 115	14.25 (20.5)	6 - 36	16.95 (5.98)	20 - 94	48.06 (19.57)	-0.01	0.008	[0,0.023]	0.003	*
		Gender	844	0 - 115	14.25 (20.5)	6 - 36	16.95 (5.98)	0 - 1	0.49 (0.5)	0.16	0.001	[-0.001,0.01]	0.310	
		Socioeconomic Status	844	0 - 115	14.25 (20.5)	6 - 36	16.95 (5.98)	3 - 20	11.88 (4.15)	0.04	0.006	[-0.001,0.023]	0.029	
		Racial or Ethnic Minority	844	0 - 115	14.25 (20.5)	6 - 36	16.95 (5.98)	0 - 1	0.51 (0.5)	-0.19	0.001	[-0.002,0.009]	0.213	
	Health-related	Everyday Cognitive Decline	835	0 - 115	14.32 (20.58)	6 - 36	16.89 (5.96)	1 - 3.5	1.34 (0.42)	0.26	0.004	[-0.002,0.03]	0.193	
		Mental Health	844	0 - 115	14.25 (20.5)	6 - 36	16.95 (5.98)	4 - 20	14.08 (3.64)	0.02	0.004	[0,0.031]	0.258	
		Physical Health	844	0 - 115	14.25 (20.5)	6 - 36	16.95 (5.98)	5 - 20	15.35 (2.97)	-0.01	0.000	[-0.003,0]	0.824	

Table S7. Study 3 FEVS single factors controlling for age, gender, race, and education level where applicable

* significant after multiple comparison correction, alpha corrected = 0.0027

Psychosocial Factor		Size	Range	Mean (Std)	Range	Mean (Std)	beta coefficient	f² (effect size)	95% CI	p value	
			FEVS		Risk Factor						
Economic Decision Making Factors	Trust in Strangers	844	0 - 18	5.25 (4.05)	0 - 1	0.45 (0.32)	0.01	0.000	[-0.001,0]	0.877	
	Trust in Friends	844	0 - 18	5.25 (4.05)	0 - 1	0.66 (0.31)	0.00	0.000	[-0.001,0.003]	0.919	
	Trust in Friends vs Strangers	844	0 - 18	5.25 (4.05)	-0.7 - 1	0.22 (0.32)	0.00	0.000	[-0.001,0]	0.950	
	Unfair Offer Rejection	844	0 - 18	5.25 (4.05)	0 - 1	0.17 (0.38)	0.02	0.000	[-0.001,0.002]	0.810	
Social and Affective Factors	Perceived Social Support	844	0 - 18	5.25 (4.05)	1 - 7	5.04 (1.4)	-0.20	0.047	[0.014,0.075]	< 0.001	*
	Cognitive Reflection	844	0 - 18	5.25 (4.05)	0 - 7	1.72 (1.57)	-0.06	0.004	[0,0.015]	0.091	
	Need to Belong	844	0 - 18	5.25 (4.05)	10 - 49	28.52 (6.73)	0.16	0.027	[0.007,0.052]	< 0.001	*
	Persuadability	844	0 - 18	5.25 (4.05)	6 - 42	14.16 (7.61)	0.28	0.092	[0.043,0.125]	< 0.001	*
	Insensitivity to Trustworthiness Cues	844	0 - 18	5.25 (4.05)	6 - 36	16.95 (5.98)	0.23	0.058	[0.021,0.088]	< 0.001	*
Socialdemographics & Health-related Moderator	Age	844	0 - 18	5.25 (4.05)	20 - 94	48.06 (19.57)	-0.39	0.183	[0.122,0.251]	< 0.001	*
	Racial or Ethnic Minority	844	0 - 18	5.25 (4.05)	0 - 1	0.51 (0.5)	0.09	0.002	[0,0.011]	0.189	
	Gender	844	0 - 18	5.25 (4.05)	0 - 1	0.49 (0.5)	-0.09	0.003	[0,0.014]	0.155	
	Socioeconomic Status	844	0 - 18	5.25 (4.05)	3 - 20	11.88 (4.15)	-0.20	0.050	[0.018,0.075]	< 0.001	*
	Physical Health	844	0 - 18	5.25 (4.05)	5 - 20	15.35 (2.97)	-0.35	0.151	[0.076,0.184]	< 0.001	*
	Mental Health	844	0 - 18	5.25 (4.05)	4 - 20	14.08 (3.64)	-0.44	0.248	[0.138,0.279]	< 0.001	*

Table S8. Study 3 FEVS interactions controlling for age, gender, race, and education level where applicable

* significant after multiple comparison correction, alpha corrected = 0.0027

Psychosocial Risk Factor	Socialdemographics & Health-related Moderator		Size	FEVS Range	FEVS Mean (Std)	Psychosocial Risk Factor Range	Psychosocial Risk Factor Mean (Std)	Socialdemographic & Health-related Moderator Range	Socialdemographic & Health-related Moderator Mean (Std)	interaction beta	f² (effect size)	95% CI	p value
Perceived Social Support	Socio-demographic	Age	844	0 - 18	5.25 (4.05)	1 - 7	5.04 (1.4)	20 - 94	48.06 (19.57)	-0.008	0.000	[-0.003,0.005]	0.133
		Gender	844	0 - 18	5.25 (4.05)	1 - 7	5.04 (1.4)	0 - 1	0.49 (0.5)	-0.154	0.001	[-0.001,0.008]	0.418
		Socioeconomic Status	844	0 - 18	5.25 (4.05)	1 - 7	5.04 (1.4)	3 - 20	11.88 (4.15)	-0.031	-0.001	[-0.001,0.009]	0.160
		Racial or Ethnic Minority	844	0 - 18	5.25 (4.05)	1 - 7	5.04 (1.4)	0 - 1	0.51 (0.5)	0.115	0.001	[-0.001,0.006]	0.552
	Health-related	Everyday Cognitive Decline	835	0 - 18	5.21 (4.04)	1 - 7	5.05 (1.41)	1 - 3.5	1.34 (0.42)	0.385	0.004	[0,0.018]	0.080
		Mental Health	844	0 - 18	5.25 (4.05)	1 - 7	5.04 (1.4)	4 - 20	14.08 (3.64)	0.008	0.001	[-0.001,0.008]	0.704
		Physical Health	844	0 - 18	5.25 (4.05)	1 - 7	5.04 (1.4)	5 - 20	15.35 (2.97)	0.061	0.007	[0,0.02]	0.031
Need to Belong	Socio-demographic	Age	844	0 - 18	5.25 (4.05)	10 - 49	28.52 (6.73)	20 - 94	48.06 (19.57)	-0.001	0.001	[0,0.007]	0.339
		Gender	844	0 - 18	5.25 (4.05)	10 - 49	28.52 (6.73)	0 - 1	0.49 (0.5)	-0.026	0.000	[-0.001,0]	0.518
		Socioeconomic Status	844	0 - 18	5.25 (4.05)	10 - 49	28.52 (6.73)	3 - 20	11.88 (4.15)	-0.004	-0.001	[-0.002,0.001]	0.421
		Racial or Ethnic Minority	844	0 - 18	5.25 (4.05)	10 - 49	28.52 (6.73)	0 - 1	0.51 (0.5)	0.019	0.000	[-0.001,0.002]	0.634
	Health-related	Everyday Cognitive Decline	835	0 - 18	5.21 (4.04)	10 - 49	28.46 (6.71)	1 - 3.5	1.34 (0.42)	-0.021	0.000	[-0.001,0.002]	0.704
		Mental Health	844	0 - 18	5.25 (4.05)	10 - 49	28.52 (6.73)	4 - 20	14.08 (3.64)	0.006	0.001	[-0.001,0.006]	0.182
		Physical Health	844	0 - 18	5.25 (4.05)	10 - 49	28.52 (6.73)	5 - 20	15.35 (2.97)	-0.006	0.001	[-0.001,0.005]	0.309
Persuadability	Socio-demographic	Age	844	0 - 18	5.25 (4.05)	6 - 42	14.16 (7.61)	20 - 94	48.06 (19.57)	0.001	0.000	[-0.002,0]	0.415

		Gender	844	0 - 18	5.25 (4.05)	6 - 42	14.16 (7.61)	0 - 1	0.49 (0.5)	-0.027	-0.001	[-0.002,0]	0.424	
		Socioeconomic Status	844	0 - 18	5.25 (4.05)	6 - 42	14.16 (7.61)	3 - 20	11.88 (4.15)	0.005	-0.001	[-0.001,0.01]	0.132	
		Racial or Ethnic Minority	844	0 - 18	5.25 (4.05)	6 - 42	14.16 (7.61)	0 - 1	0.51 (0.5)	-0.017	0.000	[-0.002,0.003]	0.621	
	Health-related	Everyday Cognitive Decline	835	0 - 18	5.21 (4.04)	6 - 42	14.07 (7.59)	1 - 3.5	1.34 (0.42)	-0.116	0.016	[0.002,0.027]	0.001	*
		Mental Health	844	0 - 18	5.25 (4.05)	6 - 42	14.16 (7.61)	4 - 20	14.08 (3.64)	0.006	0.001	[-0.001,0.007]	0.085	
		Physical Health	844	0 - 18	5.25 (4.05)	6 - 42	14.16 (7.61)	5 - 20	15.35 (2.97)	-0.008	0.002	[-0.001,0.01]	0.119	
Insensitivity to Trustworthiness Cues	Socio-demographic	Age	844	0 - 18	5.25 (4.05)	6 - 36	16.95 (5.98)	20 - 94	48.06 (19.57)	-0.001	0.001	[-0.001,0.007]	0.371	
		Gender	844	0 - 18	5.25 (4.05)	6 - 36	16.95 (5.98)	0 - 1	0.49 (0.5)	-0.032	0.001	[0,0.01]	0.466	
		Socioeconomic Status	844	0 - 18	5.25 (4.05)	6 - 36	16.95 (5.98)	3 - 20	11.88 (4.15)	0.009	0.001	[0,0.014]	0.085	
		Racial or Ethnic Minority	844	0 - 18	5.25 (4.05)	6 - 36	16.95 (5.98)	0 - 1	0.51 (0.5)	0.013	-0.001	[-0.003,0]	0.770	
	Health-related	Everyday Cognitive Decline	835	0 - 18	5.21 (4.04)	6 - 36	16.89 (5.96)	1 - 3.5	1.34 (0.42)	-0.193	0.019	[0.003,0.032]	< 0.001	*
		Mental Health	844	0 - 18	5.25 (4.05)	6 - 36	16.95 (5.98)	4 - 20	14.08 (3.64)	0.006	0.000	[-0.002,0.004]	0.198	
		Physical Health	844	0 - 18	5.25 (4.05)	6 - 36	16.95 (5.98)	5 - 20	15.35 (2.97)	-0.008	0.002	[0,0.01]	0.215	

Table S9. OAFEM Item-level Mapping on Trust in Others and Fairness Preference

#	Content	Trust or fairness related	Concept/Severity Group
1	Has someone given you poor reasons for spending your money?	Trust & Fairness	Financial Entitlement/Entitlement Expectation
2	Has someone convinced you to turn the title of your home over to them?	Trust & Fairness	Abuse of Trust/Major Theft & Scam
3	Have you thought someone was lying about how they were spending your money?	Trust	Coercion/Entitlement Expectation
4	Has someone refused to give you an accounting of spending your money?	NA	Signs of Possible Abuse/Entitlement Expectation
5	Have there been unexplained disappearances of your funds or possessions?	NA	Signs of Possible Abuse/Lesser Theft & Scams
6	Have your legal or financial documents been frequently changed (for example, the deeds of your house, your will, insurance policies or share holdings)?	Trust	Signs of Possible Abuse/Major Theft & Scam
7	Has someone persuaded you to sign any documents even though it was not in your best interest?	Trust & Fairness	Abuse of Trust/Lesser Theft & Scams
8	Has someone become the payee on your benefit check and used the money for themselves?	Trust	Theft & Scams/Major Theft & Scams
9	Has someone changed your direct deposit destination as to benefit themselves?	NA	Financial Entitlement/Major Theft & Scams
10	Have there been unusual activities in your bank accounts, for example, large withdrawals, frequent transfer of funds?	NA	Signs of Possible Abuse/Lesser Theft & Scams
11	Have there been unauthorized withdrawals from your bank account?	NA	Theft & Scams/Lesser Theft & Scams
12	Has someone tricked or pressured you into buying something that you now regret buying?	Trust & Fairness	Theft & Scams/Major Theft & Scams
13	Have you been pressured to modify your will?	NA	Sign of Possible Abuse/Major Theft & Scams
14	Has someone forced you to sign legal or financial documents?	NA	Coercion/Major Theft & Scams
15	Has someone manipulated you to give him/her larger than usual gifts (money, cars, homes)?	Trust & Fairness	Coercion/Major Theft & Scams
16	Have you been pressured to co-sign any loans?	NA	Coercion/Major Theft & Scams
17	Has someone used your money on themselves instead of for you?	NA	Abuse of Trust/Entitlement Expectation
18	Has someone borrowed money and not paid it back?	Trust	Abuse of Trust/Entitlement Expectation

19	Has someone said they were buying something for you, but it was really for their own use?	Trust & Fairness	Abuse of Trust/Lesser Theft & Scam
20	Has anyone switched some of your expensive items for cheaper ones?	NA	Theft & Scams/Major Theft & Scams
21	Has someone overcharged you for work or services that were were done poorly or never done?	Trust	Theft & Scams/Major Theft & Scams
22	Did someone take advantage of you to get a hold of your resources such as a house, car or money?	Trust	Abuse of Trust/Entitlement Expectation
23	Has someone prevented you from spending your money in order to preserve their inheritance?	Fairness	Abuse of Trust/Major Theft & Scam
24	Has someone taken your money to do something for you but never did?	Trust & Fairness	Theft & Scams/Entitlement Expectation
25	Has someone handled your money irresponsibly, for example, for gambling or illegal activities?	Trust	Theft & Scams/Lesser Theft & Scams
26	Has someone demanded money from you?	NA	Coercion/Entitlement Expectation
27	Did you let someone spend your money on themselves because you were afraid of them?	NA	Coercion/Entitlement Expectation
28	Has someone taken advantage of cultural or family expectations to get your resources?	NA	Coercion/Entitlement Expectation
29	Has someone promised you lifetime care but then did not provide it?	Trust & Fairness	Abuse of Trust/Major Theft & Scam
30	Has someone felt entitled to use your money for him/herself?	NA	Financial Entitlement/Entitlement Expectation

Table S10. FEVS-SF Item-level Mapping on Trust in Others and Fairness Preference

#	Content	Trust or fairness related
1	How worried are you about having enough money to pay for things?	NA
2	Overall, how satisfied are you with your finances?	NA
3	How satisfied are you with this money management arrangement?	NA
4	How confident are you in making big financial decisions?	Trust
5	How often do you worry about financial decisions you've recently made?	NA
6	How often do your monthly expenses exceed your regular monthly income?	NA
7	How often do you wish you had someone to talk to about financial decisions, transactions, or plans?	Trust
8	How often do you feel anxious about your financial decisions and/or transactions?	Trust and Fairness
9	How often do you feel downhearted or blue about your financial situation or decisions?	Trust

Table S11. Study 1, mapping between reported analyses and pre-registered hypotheses of single factor effects

Risk Factor			Preregistered Hypothesis
Sociodemographic factors		Socioeconomic Status	"a. Socioeconomic status and STF [susceptibility to fraud] are negatively correlated"
Health - related Factors		Physical Health	"c. Health and STF are negatively correlated"
		Mental Health	
		Everyday Cognitive Decline	"f. Cognitive abilities and STF are negatively correlated"
Psychosocial Factors	Economic Decision Making Factors	Trust in Strangers	"d. Trust in others and STF are positively correlated"
		Trust in Friends	
		Trust in Friends vs Strangers	
		Unfair Offer Rejection Rate	"e. Evaluation of fair and unfair monetary offers is associated with STF"
		Fair Offer Rejection Rate	
		Rejection Rate, Unfair Offers vs Fair Offers	
	Social and Affective Factors	Perceived Social Support	"g. Social support and STF are negatively correlated"
		Need to Belong	"h. A person's "need to belong" and STF are positively correlated"
		Persuadability	not specified in the preregistration
		Insensitivity to trustworthiness	not specified in the preregistration
		Extrinsic Affect-Improving	"j. Emotion regulation and susceptibility to fraud are negatively correlated"
		Extrinsic Affect-Worsening	
		Intrinsic Affect-Improving	
		Intrinsic Affect-Worsening	

Table S12. Study 1, planned regression analyses for single factor effects of risk factors

* significant after Bonferroni correction, alpha corrected = 0.002

Risk Factor			beta	f^2	95% CI	p value		Preregistered hypothesis
Sociodemographic factors		Socioeconomic Status	-0.03	0.001	[0,0.004]	0.128		"a. Socioeconomic status and STF [susceptibility to fraud] are negatively correlated"
Health-related Factors		Physical Health	-0.12	0.015	[0.005,0.032]	0.000	*	"c. Health and STF are negatively correlated"
		Mental Health	-0.16	0.024	[0.01,0.051]	0.000	*	
		Everyday Cognitive Decline	0.15	0.022	[0.007,0.055]	0.000	*	"f. Cognitive abilities and STF are negatively correlated"
Psycho social Factors	Economic Decision Making Factors	Trust in Strangers	0.01	0.000	[0,0.001]	0.540		"d. Trust in others and STF are positively correlated"
		Trust in Friends	-0.01	0.000	[0,0.001]	0.688		
		Trust in Friends vs Strangers	-0.02	0.000	[0,0.003]	0.241		
		Unfair Offer Rejection Rate	0.02	0.000	[0,0.004]	0.233		"e. Evaluation of fair and unfair monetary offers is associated with STF"
		Fair Offer Rejection Rate	-0.02	0.001	[0,0.002]	0.171		
		Rejection Rate, Unfair Offers vs Fair Offers	0.03	0.001	[0,0.005]	0.104		
	Social and Affective Factors	Perceived Social Support	-0.11	0.013	[0.004,0.027]	0.000	*	"g. Social support and STF are negatively correlated"
		Need to Belong	0.01	0.000	[0,0.002]	0.367		"h. A person’s “need to belong” and STF are positively correlated"
		Extrinsic Affect-Improving	0.07	0.005	[0.002,0.012]	0.000	*	"j. Emotion regulation and susceptibility to fraud are negatively correlated"
		Extrinsic Affect-Worsening	0.07	0.005	[0.001,0.021]	0.000	*	
		Intrinsic Affect-Improving	0.06	0.003	[0.001,0.009]	0.000	*	
		Intrinsic Affect-Worsening	0.14	0.019	[0.004,0.056]	0.000	*	

Table S13. Study 1, mapping between reported analyses and pre-registered hypotheses of interaction effects

Risk Factor			Preregistered Hypothesis
Health - related Factors		Physical Health * SES	"q. The relationship between socioeconomic status and STF will be lower for people who are less healthy"
		Mental Health * SES	
		Everyday Cognitive Decline * SES	"r. The relationship between socioeconomic status and STF will be lower for people with lower cognitive abilities"
Psychosocial Factors	Economic Decision Making Factors	Trust in Strangers * SES	not specified in the preregistration
		Trust in Friends * SES	
		Trust in Friends vs Strangers * SES	"n. The relationship between socioeconomic status and STF will be higher for those who trust strangers as much as their friends"
		Unfair Offer Rejection Rate * SES	"m. The relationship between socioeconomic status and STF will be lower for people who are better at evaluating fairness in others"
		Fair Offer Rejection Rate * SES	
		Rejection Rate, Unfair Offers vs Fair Offers * SES	
	Social and Affective Factors	Perceived Social Support * SES	"p. The relationship between socioeconomic status and STF will be lower for people who have lower perceived social support"
		Need to Belong * SES	"o. The relationship between socioeconomic status and STF will be lower for people who have a higher need to belong"
		Persuadability * SES	not specified in the preregistration
		Insensitivity to trustworthiness * SES	
		Extrinsic Affect-Improving * SES	
		Extrinsic Affect-Worsening * SES	
		Intrinsic Affect-Improving * SES	
		Intrinsic Affect-Worsening * SES	
Un reported			"k. The relationship between socioeconomic status and STF will be lower for those with a self-reported personal or family history of AD/ADRD"
			"l. The relationship between socioeconomic status and STF will be lower for people who are more impulsive"

Table S14. Study 1, planned regression analyses for interactions

* significant after Bonferroni correction, alpha corrected = 0.002

Risk Factor			interaction beta	f² (effect size)	95% CI	p value	Preregistered hypothesis
Health-related Factors		Physical Health * SES	0.00	0.000	[-0.002,0]	0.892	"q. The relationship between socioeconomic status and STF will be lower for people who are less healthy"
		Mental Health * SES	0.02	0.003	[0,0.011]	0.014	
		Everyday Cognitive Decline * SES	0.09	0.001	[-0.001,0.021]	0.271	"r. The relationship between socioeconomic status and STF will be lower for people with lower cognitive abilities"
Psycho social Factors	Economic Decision Making Factors	Trust in Friends vs Strangers * SES	0.14	0.000	[0,0.004]	0.268	"n. The relationship between socioeconomic status and STF will be higher for those who trust strangers as much as their friends"
		Unfair Offer Rejection Rate * SES	0.01	0.000	[-0.001,0]	0.951	"m. The relationship between socioeconomic status and STF will be lower for people who are better at evaluating fairness in others"
		Fair Offer Rejection Rate * SES	0.67	0.001	[0,0.02]	0.048	
		Rejection Rate, Unfair Offers vs Fair Offers * SES	-0.06	0.000	[-0.001,0.001]	0.503	
	Social and Affective Factors	Perceived Social Support * SES	0.06	0.003	[0,0.018]	0.036	"p. The relationship between socioeconomic status and STF will be lower for people who have lower perceived social support"
		Need to Belong * SES	0.00	0.000	[0,0.001]	0.696	"o. The relationship between socioeconomic status and STF will be lower for people who have a higher need to belong"

Table S15. Study 2, mapping between reported analyses and pre-registered hypotheses of single factor effects

Risk Factor			Preregistered Hypothesis
Sociodemographic factor		Socioeconomic Status	"a. Socioeconomic status and STF [susceptibility to fraud] are negatively correlated" (retained from Study 1)
Health - related Factors		Physical Health	"c. Health and STF are negatively correlated" (retained from Study 1)
		Everyday Cognitive Decline	"f. Cognitive abilities and STF are negatively correlated" (retained from Study 1)
Psychosocial Factors	Economic Decision Making Factors	Trust in Strangers	"d. Trust in others and STF are positively correlated" (retained from Study 1)
		Unfair Offer Rejection	"m. The relationship between socioeconomic status and STF will be lower for people who are better at evaluating fairness in others" (retained from Study 1)
	Social and Affective Factors	Perceived Social Support	"g. Social support and STF are negatively correlated" (retained from Study 1)
		Persuadability	"We also predict that STF will have positive main effects on ... gullibility, ..."
		Insensitivity to trustworthiness	
		Extrinsic Affect-Improving	"j. Emotion regulation and susceptibility to fraud are negatively correlated." (retained from Study 1)
		Extrinsic Affect-Worsening	
		Intrinsic Affect-Improving	
		Intrinsic Affect-Worsening	

Table S16. Study 2, planned ANOVA for single factor effects of risk factors

* significant after multiple comparison correction, alpha corrected = 0.0034

Risk Factor			F value	p value	Eta squared	95% CI	Shapiro's test	Levene's test	Preregistered hypothesis
Sociodemographic factor		Socioeconomic Status	1.70	0.185	0.011	[0,1]	0.000	0.222	"a. Socioeconomic status and STF [susceptibility to fraud] are negatively correlated" (retained from Study 1)
Health - related Factors		Physical Health	19.51	0.000*	0.114	[0.061,1]	0.000	0.071	"c. Health and STF are negatively correlated" (retained from Study 1)
Psycho social Factors	Economic Decision Making Factors	Trust, Strangers	4.43	0.013	0.028	[0.003,1]	0.000	0.232	"d. Trust in others and STF are positively correlated" (retained from Study 1)
		Fairness, unfair	3.29	0.039	0.021	[0.001,1]	0.000	0.637	"m. The relationship between socioeconomic status and STF will be lower for people who are better at evaluating fairness in others" (retained from Study 1)
	Social and Affective Factors	Extrinsic Affect-Improving	6.18	0.002*	0.039	[0.009,1]	0.000	0.002	"j. Emotion regulation and susceptibility to fraud are negatively correlated." (retained from Study 1)
		Extrinsic Affect-Worsening	2.44	0.089	0.016	[0,1]	0.000	0.974	
		Intrinsic Affect-Improving	6.01	0.003*	0.038	[0.008,1]	0.000	0.004	
		Intrinsic Affect-Worsening	11.52	0.000*	0.071	[0.029,1]	0.000	0.004	
		Cognitive Decline	0.43	0.650	0.003	[0,1]	0.000	0.100	"f. Cognitive abilities and STF are negatively correlated" (retained from Study 1)
		Persuadability	13.44	0.000*	0.082	[0.036,1]	0.000	0.017	"We also predict that STF will have positive main effects on ... gullibility, ..."
		Insensitivity to Trustworthiness	8.76	0.000*	0.055	[0.018,1]	0.001	0.746	
		Perceived Social Support	2.39	0.093	0.016	[0,1]	0.000	0.093	"g. Social support and STF are negatively correlated" (retained from Study 1)

Table S17. Study 2, mapping between reported analyses and pre-registered hypotheses of interaction effects

<table>
<tr><th colspan="3">Risk Factor</th><th>Preregistered Hypothesis</th></tr>
<tr><td colspan="2" rowspan="2">Health - related Factors</td><td>Physical Health * SES</td><td>"q. The relationship between socioeconomic status and STF will be lower for people who are less healthy" (retained from Study 1)</td></tr>
<tr><td>Everyday Cognitive Decline * SES</td><td>"r. The relationship between socioeconomic status and STF will be lower for people with lower cognitive abilities" (retained from Study 1)</td></tr>
<tr><td rowspan="10">Psychosocial Factors</td><td rowspan="2">Economic Decision Making Factors</td><td>Trust in Strangers * SES</td><td>not specified in the preregistration</td></tr>
<tr><td>Unfair Offer Rejection * SES</td><td>"m. The relationship between socioeconomic status and STF will be lower for people who are better at evaluating fairness in others" (retained from Study 1)</td></tr>
<tr><td rowspan="8">Social and Affective Factors</td><td>Perceived Social Support * SES</td><td>"p. The relationship between socioeconomic status and STF will be lower for people who have lower perceived social support" (retained from Study 1)</td></tr>
<tr><td>Persuadability * SES</td><td rowspan="7">not specified in the preregistration</td></tr>
<tr><td>Insensitivity to trustworthiness * SES</td></tr>
<tr><td>Extrinsic Affect-Improving * SES</td></tr>
<tr><td>Extrinsic Affect-Worsening * SES</td></tr>
<tr><td>Intrinsic Affect-Improving * SES</td></tr>
<tr><td>Intrinsic Affect-Worsening * SES</td></tr>
</table>

Table S18. Study 2, planned ANOVA for interactions

* significant after multiple comparison correction, alpha corrected = 0.0034

Risk Factor		F value	p value	Eta squared	95% CI	Shapiro's test	Levene's test	Preregistered hypothesis
Physical Health	Social Support	1.891	0.1527	0.012	[0,1]	0.000	0.232	"b) OAFEM interacts with health, with individuals with poorer health having higher OAFEM scores if they have less social support."
	Insensitivity to Trustworthines s	1.224	0.2955	0.008	[0,1]	0.005	0.746	"d) OAFEM interacts with health, with individuals with poorer health having higher OAFEM scores if they are more insensitive to trustworthiness cues."
SES household income	Fairness	0.039	0.9619	0	[0,1]	0.000	0.222	"m. The relationship between socioeconomic status and STF will be lower for people who are better at evaluating fairness in others" (retained from Study 1)
	Physical Health	3.186	0.0427	0.021	[0,1]	0.000	0.222	"q. The relationship between socioeconomic status and STF will be lower for people who are less healthy" (retained from Study 1)
	Cognitive Decline	2.377	0.0946	0.016	[0,1]	0.000	0.222	"r. The relationship between socioeconomic status and STF will be lower for people with lower cognitive abilities" (retained from Study 1)
	Social Support	0.291	0.748	0.002	[0,1]	0.000	0.232	"c) OAFEM interacts with SES/household income (see below; HHI) and social support: individuals with lower SES/HHI having higher OAFEM scores if they have less social support."
Gender	Social Support	0.649	0.5234	0.004	[0,1]	0.000	0.232	"a) OAFEM interacts with gender, with women having higher OAFEM scores if they have less social support."

Table S19. Study 3, mapping between reported analyses and pre-registered hypotheses of single factor effects

Risk Factor		Preregistered Hypothesis
Economic Decision Making Factors	Trust in Strangers	"We predict that psychosocial variables tied to trust, fairness, social support, cognitive reflection, and need to belong will be associated with risk for financial exploitation."
	Trust in Friends	
	Trust in Friends vs Strangers	
	Unfair Offer Rejection	
Social and Affective Factors	Perceived Social Support	
	Cognitive Reflection	
	Need to Belong	
	Persuadability	
	Insensitivity to Trustworthiness Cues	
Socialdemographics & Health-related Moderator	Racial or Ethnic Minority	not specified in the preregistration (main effect follow-ups after insignificant interactions)
	Gender	
	Socioeconomic Status	
	Everyday Cognitive Decline	
	Mental Health	
	Physical Health	

Table S20. Study 3, planned ANOVA for single factor effects of risk factors

* significant after multiple comparison correction, alpha corrected = 0.0029

Psychosocial Risk Factor	F value	p value	Eta squared	95% CI	Shapiro's test	Levene's test	Preregistered hypothesis
Trust, Strangers	0.30	0.742	0.001	[0,1]	0.000	0.360	"We predict that psychosocial variables tied to trust, fairness, social support, cognitive reflection, and need to belong will be associated with risk for financial exploitation."
Trust, Friends	0.24	0.789	0.001	[0,1]	0.000	0.776	
Trust, Friends VS Strangers	0.19	0.829	0.001	[0,1]	0.000	0.635	
Persuadability	66.50	0.000*	0.242	[0.184,1]	0.000	0.000	
Insensitivity to Trustworthiness	44.03	0.000*	0.174	[0.121,1]	0.009	0.494	
Fairness, unfair	0.01	0.992	0	[0,1]	0.000	0.992	
Perceived Social Support	7.62	0.001*	0.035	[0.01,1]	0.000	0.016	
Cognitive Reflection	6.66	0.001*	0.031	[0.008,1]	0.000	0.003	
Need to Belong	41.20	0.000*	0.165	[0.113,1]	0.187	0.738	

Table S21. Study 3, mapping between reported analyses and pre-registered hypotheses of interaction effects

Psychosocial Risk Factor	Socialdemographics & Health-related Moderator		Preregistered Hypothesis
Perceived Social Support	Sociodemographic	Age	"We also predict that these relationships will be moderated by sociodemographic factors such as age, socioeconomic status (SES), and race/ethnicity and also health-related variables (cognitive, psychological, and physical)."
		Gender	
		Socioeconomic Status	
		Racial or Ethnic Minority	
	Health-related	Everyday Cognitive Decline	
		Mental Health	
		Physical Health	
Need to Belong	Sociodemographic	Age	
		Gender	
		Socioeconomic Status	
		Racial or Ethnic Minority	
	Health-related	Everyday Cognitive Decline	
		Mental Health	
		Physical Health	
Persuadability	Sociodemographic	Age	
		Gender	
		Socioeconomic Status	
		Racial or Ethnic Minority	
	Health-related	Everyday Cognitive Decline	
		Mental Health	
		Physical Health	
Insensitivity to Trustworthiness Cues	Sociodemographic	Age	
		Gender	
		Socioeconomic Status	
		Racial or Ethnic Minority	
	Health-related	Everyday Cognitive Decline	
		Mental Health	
		Physical Health	

Table S22. Study 3, planned ANOVA for interactions

* significant after multiple comparison correction, alpha corrected = 0.0029

Psychosocial Risk Factor	Socialdemographics & Health-related Moderator	F value	p value	Eta squared	95% CI	Shapiro's test	Levene's test	Preregistered hypothesis
Trust in Stranger	Age	1.26	0.284	0.006	[0,1]	0.000	0.360	"We also predict that these relationships will be moderated by sociodemographic factors such as age, socioeconomic status (SES), and race/ethnicity and also health-related variables (cognitive, psychological, and physical)."
	Gender	2.63	0.074	0.013	[0,1]	0.000	0.360	
	Minority	0.93	0.395	0.004	[0,1]	0.000	0.360	
	SES	1.65	0.194	0.008	[0,1]	0.000	0.360	
	Cognitive Decline	0.45	0.639	0.002	[0,1]	0.000	0.371	
	Physical Health	2.80	0.062	0.013	[0,1]	0.000	0.360	
	Mental Health	0.19	0.825	0.001	[0,1]	0.000	0.360	
Persuadability	Age	2.67	0.071	0.013	[0,1]	0.000	0.000	
	Gender	1.92	0.148	0.009	[0,1]	0.000	0.000	
	Minority	0.15	0.859	0.001	[0,1]	0.000	0.000	
	SES	9.60	0.000*	0.044	[0.016,1]	0.000	0.000	
	Cognitive Decline	0.27	0.767	0.001	[0,1]	0.000	0.000	
	Physical Health	2.44	0.089	0.012	[0,1]	0.000	0.000	
	Mental Health	18.46	0.000*	0.082	[0.043,1]	0.000	0.000	
Insensitivity to Trustworthiness	Age	1.05	0.352	0.005	[0,1]	0.010	0.494	
	Gender	0.83	0.438	0.004	[0,1]	0.014	0.494	
	Minority	1.23	0.295	0.006	[0,1]	0.016	0.494	
	SES	1.02	0.363	0.005	[0,1]	0.015	0.494	
	Cognitive Decline	0.86	0.425	0.004	[0,1]	0.025	0.473	
	Physical Health	1.42	0.244	0.007	[0,1]	0.049	0.494	
	Mental Health	0.38	0.686	0.002	[0,1]	0.025	0.494	
Social Support	Age	0.36	0.701	0.002	[0,1]	0.000	0.016	
	Gender	1.54	0.216	0.007	[0,1]	0.000	0.016	

	Minority	0.84	0.432	0.004	[0,1]	0.000	0.016	
	SES	4.24	0.015	0.02	[0.002,1]	0.000	0.016	
	Cognitive Decline	1.96	0.142	0.009	[0,1]	0.000	0.016	
	Physical Health	0.23	0.794	0.001	[0,1]	0.000	0.016	
	Mental Health	3.31	0.038	0.016	[0,1]	0.000	0.016	
Cognitive Reflection	Age	0.05	0.949	0	[0,1]	0.000	0.003	
	Gender	6.14	0.002*	0.029	[0.006,1]	0.000	0.003	
	Minority	1.72	0.181	0.008	[0,1]	0.000	0.003	
	SES	0.79	0.455	0.004	[0,1]	0.000	0.003	
	Cognitive Decline	0.91	0.403	0.004	[0,1]	0.000	0.011	
	Physical Health	1.18	0.309	0.006	[0,1]	0.000	0.003	
	Mental Health	2.15	0.118	0.01	[0,1]	0.000	0.003	
Need to Belong	Age	1.09	0.338	0.005	[0,1]	0.273	0.738	
	Gender	2.36	0.096	0.011	[0,1]	0.258	0.738	
	Minority	0.22	0.805	0.001	[0,1]	0.458	0.738	
	SES	1.74	0.177	0.008	[0,1]	0.577	0.738	
	Cognitive Decline	0.07	0.932	0	[0,1]	0.212	0.784	
	Physical Health	0.41	0.667	0.002	[0,1]	0.272	0.738	
	Mental Health	4.60	0.011	0.022	[0.003,1]	0.523	0.738	

Figure S1. correlation matrix of the Trust Game measures and Two-Factor Gullibility Scale measures

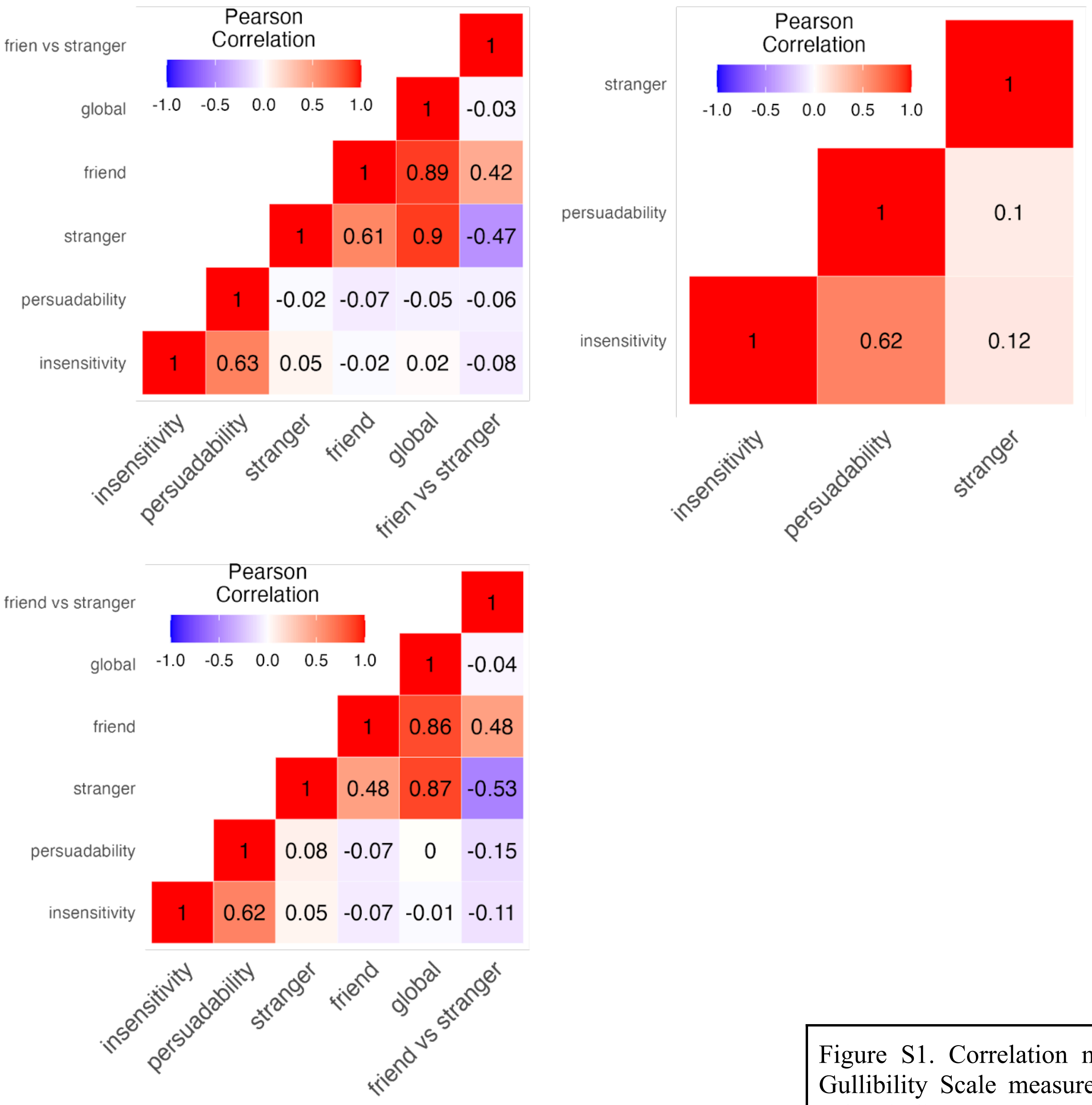

Figure S1. Correlation matrix of the Trust Game measures and Two-Factor Gullibility Scale measures. Red color indicates positive correlation and blue color indicates negative correlation. The numbers in each cell is pairwise Pearson's r.
