# Supplementary material for: Psychological, social, and health-related factors predict risk for financial exploitation
Source: Commun Psychol. 2025 Jun 5;3:88. doi: 10.1038/s44271-025-00266-x (PMC12141040; doi:10.1038/s44271-025-00266-x)
Supplement: Supplementary file 3 — Reporting Summary [file 44271_2025_266_MOESM3_ESM.pdf]

nature portfolio

Corresponding author(s): David V. Smith

Last updated by author(s): Feb 24, 2025

Reporting Summary

Nature Portfolio wishes to improve the reproducibility of the work that we publish. This form provides structure for consistency and transparency in reporting. For further information on Nature Portfolio policies, see our Editorial Policies and the Editorial Policy Checklist.

Statistics

For all statistical analyses, confirm that the following items are present in the figure legend, table legend, main text, or Methods section.

n/a	Confirmed	
☐	☒	The exact sample size (*n*) for each experimental group/condition, given as a discrete number and unit of measurement
☐	☒	A statement on whether measurements were taken from distinct samples or whether the same sample was measured repeatedly
☐	☒	The statistical test(s) used AND whether they are one- or two-sided<br>*Only common tests should be described solely by name; describe more complex techniques in the Methods section.*
☐	☒	A description of all covariates tested
☐	☒	A description of any assumptions or corrections, such as tests of normality and adjustment for multiple comparisons
☐	☒	A full description of the statistical parameters including central tendency (e.g. means) or other basic estimates (e.g. regression coefficient) AND variation (e.g. standard deviation) or associated estimates of uncertainty (e.g. confidence intervals)
☐	☒	For null hypothesis testing, the test statistic (e.g. *F*, *t*, *r*) with confidence intervals, effect sizes, degrees of freedom and *P* value noted<br>*Give P values as exact values whenever suitable.*
☒	☐	For Bayesian analysis, information on the choice of priors and Markov chain Monte Carlo settings
☒	☐	For hierarchical and complex designs, identification of the appropriate level for tests and full reporting of outcomes
☐	☒	Estimates of effect sizes (e.g. Cohen's *d*, Pearson's *r*), indicating how they were calculated

Our web collection on statistics for biologists contains articles on many of the points above.

Software and code

Policy information about availability of computer code

Data collection	All data were collected using Qualtrics panels
Data analysis	All analyses were performed in R Statistical Software (v 4.3.0, R Core Team, 2023). Robust regressions were performed using ‘rlm’ and its default settings of the MASS R package (v 7.3.60; Venables & Ripley, 2022). We adopted a robust F-test (i.e. a Wald test for multiple coefficients,) to compute the p value of a correlation coefficient in robust regression using ‘f.robftest’ and its default settings of the sfsmisc R package (version 1.1-16; Maechler, 2023).

For manuscripts utilizing custom algorithms or software that are central to the research but not yet described in published literature, software must be made available to editors and reviewers. We strongly encourage code deposition in a community repository (e.g. GitHub). See the Nature Portfolio guidelines for submitting code & software for further information.

Data

Policy information about availability of data

All manuscripts must include a data availability statement. This statement should provide the following information, where applicable:

- Accession codes, unique identifiers, or web links for publicly available datasets
- A description of any restrictions on data availability
- For clinical datasets or third party data, please ensure that the statement adheres to our policy

The data and zip code-based median household income codebook are available on https://osf.io/wxy2v/?view_only=edf03685a6644ac9aed283df4c73219b.

Research involving human participants, their data, or biological material

Policy information about studies with human participants or human data. See also policy information about sex, gender (identity/presentation), and sexual orientation and race, ethnicity and racism.

Reporting on sex and gender	Gender was collected using participant self-reports and is not the focus of the current study. Gender characteristics of each study can be found in the manuscript Table 2.
Reporting on race, ethnicity, or other socially relevant groupings	Race and ethnicity were collected using participant self-reports and are not the focus of the current study. Race and ethnicity characteristics of each study can be found in the manuscript Table 2.
Population characteristics	Study 1: adults of age 50 and above, mean = 62.48(7.89)<br>Study 2: adults of age 50 and above, mean = 61.78 (7.95)<br>Study 3: adults of age 20 and above, mean = 48.06 (19.57)
Recruitment	Participants were recruited directly by Qualtrics panels.
Ethics oversight	Temple University's Institutional Review Board

Note that full information on the approval of the study protocol must also be provided in the manuscript.

Field-specific reporting

Please select the one below that is the best fit for your research. If you are not sure, read the appropriate sections before making your selection.

☐ Life sciences ☒ Behavioural & social sciences ☐ Ecological, evolutionary & environmental sciences

For a reference copy of the document with all sections, see nature.com/documents/nr-reporting-summary-flat.pdf

Behavioural & social sciences study design

All studies must disclose on these points even when the disclosure is negative.

Study description	quantitative survey study
Research sample	Study 1: Philadelphia Metropolitan Area adults of age 50 or above<br>Study 2: Pennsylvania Region adults of age 50 or above<br>Study 3: The United States adults of age 20 or above
Sampling strategy	All participants were recruited by Qualtrics panels. The samples were projected to be evenly split based on household income, gender, and education.
Data collection	All data were collected using Qualtrics panels.
Timing	Study 1: 2/4 - 2/29, 2020<br>Study 2: 4/16 - 5/21, 2020<br>Study 3: 7/12 - 9/10, 2021
Data exclusions	Data from participants who failed Qualtrics' internal quality check (e.g., anyone completing in less than half of the median completion time of the "soft launch" during which 10% of the target sample size was collected for Qualtrics' reviewing purposes) and attention checks (e.g., asking participants to "select the 'strongly disagree'") were excluded from a study directly by Qualtrics before sending the final data to us. In each specific analysis we also excluded participants whose data needed for the analysis was not collected due to Qualtrics technical error.
Non-participation	All participants were recruited by Qualtrics panels. Qualtrics did not provided non-participation information.
Randomization	n/a

Reporting for specific materials, systems and methods

We require information from authors about some types of materials, experimental systems and methods used in many studies. Here, indicate whether each material, system or method listed is relevant to your study. If you are not sure if a list item applies to your research, read the appropriate section before selecting a response.

Materials & experimental systems

n/a	Involved in the study
☒	☐ Antibodies
☒	☐ Eukaryotic cell lines
☒	☐ Palaeontology and archaeology
☒	☐ Animals and other organisms
☒	☐ Clinical data
☒	☐ Dual use research of concern
☒	☐ Plants

Methods

n/a	Involved in the study
☒	☐ ChIP-seq
☒	☐ Flow cytometry
☒	☐ MRI-based neuroimaging

Plants

Seed stocks

n/a

Novel plant genotypes

n/a

Authentication

n/a
